# How useful are malaria risk maps at the country level? Perceptions of decision-makers in Kenya, Malawi and the Democratic Republic of Congo

**DOI:** 10.1186/s12936-020-03425-z

**Published:** 2020-10-02

**Authors:** Ludovica Ghilardi, George Okello, Linda Nyondo-Mipando, Chawanangwa Mahebere Chirambo, Fathy Malongo, Jenna Hoyt, Jieun Lee, Yovitha Sedekia, Justin Parkhurst, Jo Lines, Robert W. Snow, Caroline A. Lynch, Jayne Webster

**Affiliations:** 1 Department of Disease Control, London School of Hygiene and Tropical Medicine, London, UK; 2 Kinshasa School of Public Health, University of Kinshasa, Mont Amba/Lemba, BP 11850 Kin I, Kinshasa, Democratic Republic of Congo; 3 Department of Infectious Disease Epidemiology, London School of Hygiene and Tropical Medicine, London, UK; 4 London School of Economics and Political Science, Houghton Street, London, WC2A 2AE UK; 5 Kenya Medical Research Institute-Wellcome Trust Research Programme, P.O. Box 43640-00100, Nairobi, Kenya; 6 Centre for Tropical Medicine and Global Health, Nuffield Department of Clinical Medicine, University of Oxford, OX3 7LJ Oxford, UK; 7 World Vision UK, 1rb, 11 Belgrave Rd, Pimlico, London, SW1V 1RB UK; 8 Mwanza Intervention Trials Unit (MITU)/ National Institute for Medical Research (NIMR)- Mwanza Research Centre, P.O BOX 11936, Isamilo road, Mwanza, Tanzania; 9 Department of Health Systems and Policy, College of Medicine, University of Malawi, Blantyre, Malawi

**Keywords:** Risk maps, Modelled *Pf*PR maps, Targeting, National Malaria Control Programme, Kenya, Malawi, Democratic Republic of Congo

## Abstract

**Background:**

Declining malaria prevalence and pressure on external funding have increased the need for efficiency in malaria control in sub-Saharan Africa (SSA). Modelled *Plasmodium falciparum* parasite rate (*Pf*PR) maps are increasingly becoming available and provide information on the epidemiological situation of countries. However, how these maps are understood or used for national malaria planning is rarely explored. In this study, the practices and perceptions of national decision-makers on the utility of malaria risk maps, showing prevalence of parasitaemia or incidence of illness, was investigated.

**Methods:**

A document review of recent National Malaria Strategic Plans was combined with 64 in-depth interviews with stakeholders in Kenya, Malawi and the Democratic Republic of Congo (DRC). The document review focused on the type of epidemiological maps included and their use in prioritising and targeting interventions. Interviews (14 Kenya, 17 Malawi, 27 DRC, 6 global level) explored drivers of stakeholder perceptions of the utility, value and limitations of malaria risk maps.

**Results:**

Three different types of maps were used to show malaria epidemiological strata: malaria prevalence using a *Pf*PR modelled map (Kenya); malaria incidence using routine health system data (Malawi); and malaria prevalence using data from the most recent Demographic and Health Survey (DRC). In Kenya the map was used to target preventative interventions, including long-lasting insecticide-treated nets (LLINs) and intermittent preventive treatment in pregnancy (IPTp), whilst in Malawi and DRC the maps were used to target in-door residual spraying (IRS) and LLINs distributions in schools. Maps were also used for operational planning, supply quantification, financial justification and advocacy. Findings from the interviews suggested that decision-makers lacked trust in the modelled *Pf*PR maps when based on only a few empirical data points (Malawi and DRC).

**Conclusions:**

Maps were generally used to identify areas with high prevalence in order to implement specific interventions. Despite the availability of national level modelled *Pf*PR maps in all three countries, they were only used in one country. Perceived utility of malaria risk maps was associated with the epidemiological structure of the country and use was driven by perceived need, understanding (quality and relevance), ownership and trust in the data used to develop the maps.

## Background

Declining malaria prevalence [1–3] and malaria mortality [4], pressure on external funding and renewed interest in malaria elimination [5, 6] have highlighted the need for increased efficiency of malaria control in sub-Saharan Africa (SSA) [7–9]. The Global Technical Strategy for Malaria 2016–2030 calls for a more evidence-driven allocation of resources and tailored approach to malaria control [5, 10].

In the last 10–15 years, maps showing the proportion of individuals infected at a given point in time are increasingly replacing or becoming available alongside qualitative, eco-climatic risk and routine data maps across a number of countries in SSA [11]. Modelled *Pf*PR maps are developed by assembling community-based malaria parasite prevalence (*Pf*PR) data from the general population or specific population surveys, such as school based children [12–16], and used within geostatistical models to provide estimates of infection prevalence at unsampled locations [17]. In contexts lacking complete, good quality routine health data, modelled *Pf*PR maps provide an indication of the epidemiological characteristics of malaria transmission sub-nationally. In their application to national malaria control, the assumption is that these estimates of sub-national malaria risk can be used to prioritize and target interventions leading to a more appropriate allocation of resources and more efficient prevention and response [8].

Maps have long been used as evidence by national stakeholders to inform policy priorities, strategies and interventions [3, 18], they represent one form of relevant evidence for health decision-making. More broadly, the literature explores the use of evidence in health planning and indicates there are multiple conceptualizations around *evidence* and *use of evidence* [19, 20]. Some articles focus on scientific *evidence* as derived from randomized clinical trials [21, 22], while others refer to a more general definition of evidence which includes information and data [23]. Literature on the quality and use of routine health data, surveillance data and survey data at national and district levels in low and middle-income countries (LMIC) is expanding [23–27], as is the use of health information in humanitarian settings [28]. Studies looking specifically at the use of spatially defined health data and maps to plan interventions are less common. There remains a need to understand how evidentiary tools like malaria risk maps, showing prevalence of parasitaemia or incidence of illness, are understood and utilized in practice by policy makers and implementers. This study considered the use of spatially aggregated or mapped data as a form of *evidence* for decision-making on malaria interventions and strategies, and explored the *use* of malaria risk maps as defined by malaria policy-makers and key stakeholders.

This study was part of the evaluation of the Information for malaria (INFORM) and LINK-Data for malaria decision-making (LINK) projects, which supported 14 malaria endemic countries in SSA to develop prevalence modelled risk maps and epidemiological profiles of malaria. The aim of this study was to explore the practises and perceptions of National Malaria Control Programmes (NMCPs) staff and other malaria control stakeholders on the use of malaria risk maps, in prioritization and targeting of interventions.

## Methods

### Study site and context

The study was conducted between April 2017 and June 2018 in Kenya, Malawi and the Democratic Republic of Congo (DRC). The study sites were limited to the cities where national level stakeholders are based (Nairobi, Lilongwe, Blantyre and Kinshasa). Interviews with global stakeholders were conducted in Nairobi, Dakar (during the Multilateral Initiative on Malaria) or remotely.

Malaria is endemic across the three countries with a predominance of *Plasmodium falciparum* infection. According to the World Health Organization (WHO) guidelines, all three countries are in the malaria control phase [29]. Malaria epidemiology, decision-making structures and policies for control in each country are presented in Tables 1 and 2.

**Table 1:** Malaria epidemiology, decision making structures and policies for control

	Kenya	Malawi	DRC
Epidemiology			
Overview	High variability in malaria parasite prevalence across the country, with endemic counties around Lake Victoria and on the coast, epidemic-prone counties in the highland areas, seasonal counties and low risk counties around Nairobi [51–53]	Relatively homogeneous prevalence of malaria with higher burden along Lake Malawi in the Central and Southern regions [54]	Homogeneous hyperendemic to holoendemic malaria transmission across the country, with the exception of the mountainous area in the eastern provinces (0.2% of the population), and in the capital city Kinshasa [55, 56]
Number of estimated cases in 2018 (GMR2019)	3.6 M	3.8 M	26.8 M
Number of estimated deaths in 2018 (GMR2019)	12,416	6678	44,615
Main vectors	Predominance of *An. arabiensis* and *An. gambiae* s.s	Predominance of *An. gambiae*, and minority of *An. arabiensis* and *An. funestus*	Predominance of *An. gambiae* and *An. funestus*. In addition presence of *An. moucheti*, *An. nili*
Decision-making for malaria control			
Administrative levels of decision-making	Since 2010 Kenya has a decentralized system of 47 counties. The counties are assigned the service delivery functions while the national government provides national referral, policy guidelines, capacity building and technical assistance	Malawi is divided into three regions and 28 districts (local government units), which are further divided into Traditional Authorities ruled by a chief Policies are defined at national level; districts have technical support and monitoring functions	The country reorganised the province level in late 2015 increasing the number of provinces from 11 to 26 Policies are determined at national level. Health directorates, present in the 26 provinces, perform functions of technical support and monitoring. Under the health directorates there are 65 health districts and 515 Health zones. The Health Zone is the operational unit for planning and implementation of the national health policy
Dates when NMCPs was established	2000	1984	1998
National Malaria Strategic Plans (post-RBM)	2001–2010 2009–2018 2019–2023	1990–1994 2001–2005 2005–2010 2011–2016 2017–2022	2002–2006 2007–2011 Replaced by 2009–2013 NMSP (in line with RBM targets) Replaced by 2011–2015 NMSP aligned with the broader health sector strategic plan 2016–2020
GF and PMI support start dates	GF: 2002 PMI: 2008	GF: 2003 PMI: 2007	GF: 2003 PMI: 2011
Policies			
LLINs policies	LLINs are delivered through mass and routine distribution, including at ANC and Child Welfare Clinics, in the 23 endemic and epidemic-prone and the 13 malaria-prone counties	LLINs are delivered through mass and routine distribution at ANC and implemented universally	LLINs are delivered through mass and routine distribution at ANC and implemented universally
IPTp policies	IPTp 3 plus is delivered at routine ANC visits and implemented in the 14 lake and coastal endemic counties	IPTp 3 plus is delivered at routine ANC visits and implemented universally	IPTp 3 plus is delivered at routine ANC visits and implemented universally
IRS policies	The NMCP targets spraying in the lake-endemic counties of western Kenya (7 counties)	The NMCP targets spraying according to level of risk and budget availability (along Lake Malawi and in the southern districts)	The NMCP targets spraying according to level of risk and budget availability

**Table 2:** National Malaria Control Programme (NMCP) role and structure

Role	The programme, under the responsibility of the Ministry of Health, defines and leads the strategy of prevention and control of malaria at the national level. The NMCP is responsible for ensuring compliance with the malaria prevention and treatment national guidelines
National structure	Despite slight variations, the NMCPs are generally composed of a number of divisions including: case management; vector control; epidemiology and surveillance; monitoring and evaluation; research; finance, procurement and supply. The NMCP collaborates with partners, at national and international levels, through formal technical working groups (TWGs) and informal structures, and supports and supervises the implementation of malaria control interventions at national and district (or sub-county) level. Malaria policies are generally defined at national level
Sub-national structure	At district (or sub-county) level, a malaria officer from the NMCP is often in charge of the support and supervision of malaria control activities at all levels of health facilities, and at the community level for the delivery of interventions including long-lasting insecticidal nets (LLINs) distributions, indoor residual spraying (IRS), intermittent preventive treatment in pregnancy (IPTp) and case management

### Study design

This study was embedded within the internal evaluation of the LINK-Data for malaria decision-making project with the aim to assess the contribution of the INFORM and LINK epidemiological profiles, data and maps to malaria decision-making in SSA. The analytical framework for the evaluation was adapted from the Research Impact Framework of Kuruvilla et al. [30] and provided the basis for the analysis of how malaria data were used for decision-making.

This study was a narrative synthesis of the findings from a document review of national malaria strategic plans (NMSPs), and in-depth interviews (IDIs) with national level malaria control decision-makers and stakeholders. The document review sought evidence of the use of risk maps in national malaria strategic plans. The review was supplemented with IDIs and aimed to elucidate practises and perceptions of stakeholders on the utility of risk maps in their decision-making, in particular, how these maps affect prioritization and targeting of interventions, along with the reasoning behind their decisions.

Prioritization and targeting are often difficult to disentangle and in fact in practice, one inevitably implies the other, but in general prioritization refers to the type of intervention or the type of delivery method that should be selected for a given geographical area [31]; where as targeting is about which geographical areas and/or sub-populations should the intervention and delivery method be deployed in [32–34].

### Document review

The latest NMSPs were reviewed for each of the three countries to identify the type of malaria risk maps included (e.g. eco-climatic, based on malaria cases routine data, or based on parasite prevalence surveys) in the strategies and the reason why the maps were used. Reasons for the use of maps were extracted from each document and analysed by examining the content of text referring to the maps across the entire document.

### Stakeholder interviews

IDIs were conducted with 64 stakeholders purposively selected across the three countries and among experts at global level: Kenya 14, Malawi 17, DRC 27, global stakeholders 6 (Table 3). Stakeholders interviewed were from the NMCPs, Ministry of health (MoH) at national level, statistical and pharmaceutical governmental bodies, United Nation agencies (UN), donors, Non-Governmental Organizations (NGOs), and researchers. Stakeholder designation within these categories are not presented by country, to maintain anonymity, but for NMCP designations included director, vector control, monitoring, evaluation and surveillance, research, case management, and malaria in pregnancy (MiP) units; for MoH Health Information System and policies units; for government bodies statistical office and national pharmacies; UN agencies included WHO and UNICEF; donors included the President’s Malaria Initiative (PMI), Global Fund (GF) and the UK Department for International Development (DFID); NGOs were both national and international and researchers included epidemiologists and entomologists.

**Table 3:** Participants by role and country

	Kenya	Malawi	DRC	Global
NMCP, MoH and other government officers	4	5	9	/
Partners officers (UN agencies, donors, NGOs officers)	8	8	16	5
Researchers	2	4	2	1
Total	14	17	27	6

Interviews were conducted using theme guides to explore the following: malaria data produced, data accessibility, data use, perception of the quality of malaria data available, type of maps produced and used, type of maps and purpose of use, use for prioritization and targeting, collaboration among the stakeholders, and examples of decisions driven by data and epidemiological maps.

Stakeholders were invited to an interview at a time and place of their convenience. Interviews were conducted by LG in collaboration with national co-investigators (GO, LNM, CMC, FM) in English or French, according to the official language and were audio-recorded where consent was provided. Detailed field notes were taken where consent to record the interview was declined.

### Data management and analysis

Interviews were transcribed, translated into English and imported into NVivo software (QSR international) Version 11 for coding and analysis. The transcripts were coded according to four levels of analysis: (1) type of maps: data used in existing maps; (2) use of maps: by stakeholders, at which level, for what purpose, non-use of maps; (3) value and perception of maps: usefulness of the data, trust in the data, the value of the maps; (4) suggestions for and criticisms of the maps: production, dissemination and future development of maps. Additional themes and sub-themes that emerged from the data were added to the coding framework inductively and further explored using content analysis.

During data analysis, all stakeholders were assigned an anonymous code. Quotes were identified using labels including: role (NMCP, MoH, researcher, partner), country (Kenya, Malawi, DRC, global) and a consecutive number. Findings were validated by stakeholders (NMCP representatives and researchers) during a three-day workshop in September 2018. The main findings of the study were presented, discussed and validated with national co-investigators on day 1, and with NMCP representatives on day 2. Adjustments were made based on feedback from workshop participants, and on day 3 the findings were presented to an open audience, including international malaria experts.

## Results

The use of malaria risk maps in the National Malaria Strategic Plans (NMSPs) is presented and the perceptions of stakeholders about the utiliy and limitation of the maps are explored in further depth.

### Risk maps included in the National Malaria Strategic Plans

**Fig. 1:**
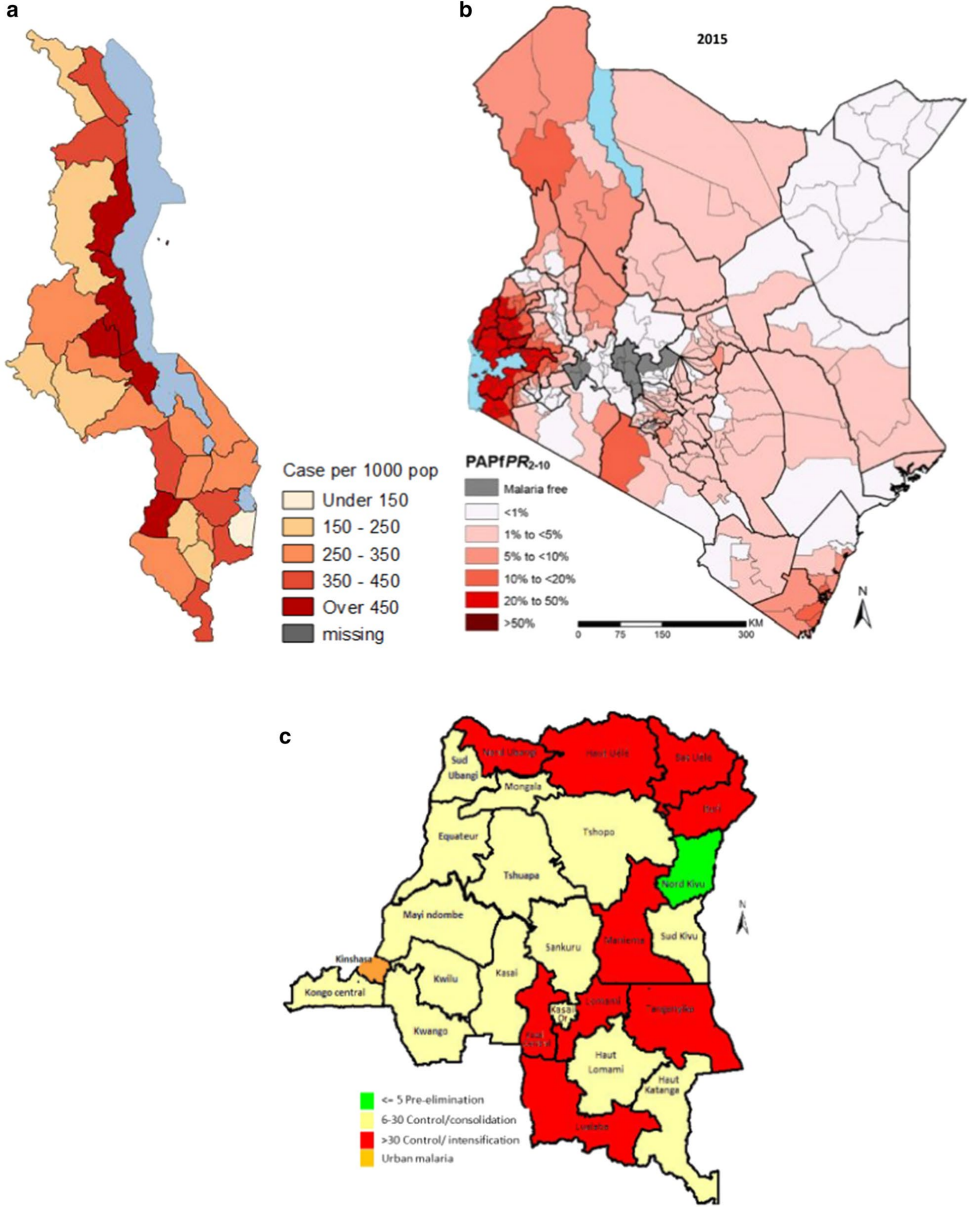
Malaria risk maps utilized in the most recent NMSPs in Malawi (**a**), Kenya (**b**), and DRC (**c**)

In all three NMSPs, malaria risk maps showing the distribution of infection and, consequently, the risk of being infected were included. However, each country used a different type of malaria risk map, based on different kinds of data: a modelled *Pf*PR map in Kenya, an incidence map based on routine data in Malawi and a prevalence map based on the Demographic Health Survey (DHS) data in DRC (Fig. 1). Although the three maps were developed using different methods and types of data, they were used in the NMSPs for the same purpose, that of showing epidemiological strata and identifying high risk areas where interventions would be implemented.

In the Kenya NMSP 2019–2023 [35] a panel of four maps were included to show and describe the malaria prevalence levels and changes over time (same map updated by different year- 2000, 2005, 2010, 2015). Kenya presented seven strata of the *Pf*PR in 2 to 10 year old children: malaria free, < 1%, 1% to 5%, 5% to 10%, 10% to 20%, 20 to 40%, > 40%. The strategy indicated that each vector control intervention (LLINs distribution, larval source control and IRS) would be deployed in specific areas according to the stratification [35].

The Malawian NMSP 2017–2022 [36] includes a panel of four maps from 2011 to 2015 to illustrate the evolution of risk according to the Annual Parasite Incidence and the number of cases per 1000 population by each district (divided into 5 categories: under 150, 150–250, 250–350, 350–450, over 450). According to the strategy, interventions would be allocated universally, with the exception of IRS, which would be implemented in high burden districts or areas [36].

The Congolese NMSP 2016–2020 [37] included one map that showed the stratification of the *Pf*PR in < 5 years old children in four strata: pre-elimination in North Kivu (*Pf*PR < 5%), control/consolidation (*Pf*PR 6–30%), control/intensification (*Pf*PR > 30%) and urban malaria (in Kinshasa). The strategy stated that all interventions would be allocated universally, with the exception of IRS, which would be implemented in urban and pre-elimination areas, and LLINs distribution in schools in the tropical areas [37].

### Perceptions of the stakeholders—what drives the use of malaria risk maps

The analysis of stakeholder interviews focused on two main thematic areas: types of use of risk maps—including strategies, prioritization, targeting and operational planning, and the drivers of the use of risk maps—including perception of value and limitations of the maps by malaria stakeholders.

### Use of risk maps

The information derived from the interviews matched and enriched that from the NMSPs. Risk maps were primarily used for strategic planning, in particular to aid in the selection of geographic areas or population sub-groups for delivery of an intervention (targeting); however, operational planning and advocacy were also identified as important uses.

### Prioritization and targeting

Selection of interventions and geographic areas.

Intervention choices were based on evidence from efficacy and effectiveness studies, as well as WHO Global Malaria Programme (GMP) guidelines. Across the three countries, treatment was widely prioritized over prevention as it was perceived as a tool for ‘saving lives’ as opposed to preventing infection. Stakeholders, including NMCPs and donors, reported that interventions were prioritized based on their perceived efficacy, which primarily meant ensuring the availability of commodities. Anti-malarials and diagnostics for case management were the first priority, followed by LLINs and IPTp for prevention. This prioritization was also seen in the Global Fund proposals reviewed in each country, where the majority of the requested budget was for commodities: artemisinin-based combination therapies (ACTs), rapid diagnostic tests (RDTs) and LLINs [38, 39]. This was confirmed in conversations with the NMCP officers who oversee Global Fund grants interviewed.

“*You have to make sure that lifesaving interventions are taken care of fully… there was no question… there was no debate about that. There have to be diagnostics, there have to be medicines. That’s number one, because we have to save people from dying. Then the second thing was prevention, accessibility to nets*.” (NMCP officer 1, Kenya)

“*Of course commodities [for case management] comes first. Next is the nets*.” (NMCP officer 6, Malawi).

The consideration of the geographical areas where interventions would be implemented was based on maps. Targeting was applied to all preventative interventions (LLINs, IPTp, IRS) in the case of Kenya; while limited to IRS, or LLINs in schools, or IPTp at the community level in the case of Malawi and DRC. In Kenya, the delivery of LLINs and IPTp was only implemented in the 16 endemic counties of the Lake and Coast regions (out of 47 counties in total) and IRS was implemented in two counties (Homa Bay and Migori). In DRC and Malawi, LLINs and IPTp were delivered in the entire country through using a universal coverage approach, however maps were utilized to identify high burden areas where additional interventions or delivery sites were appropriate, such as LLINs distributed in schools and the delivery of IPTp at the community level in DRC (Table 4) and IRS in Malawi. Specifically, IRS in Malawi was scaled up from one to seven highly endemic districts in 2012, while in DRC, IRS was implemented by mining companies in the areas where they were operating.

**Table 4:** Type and use of malaria risk map included in the most recent National Malaria Strategic Plan by country

Type of risk map	Source of data	Map resolution	Main use of the map in the NMSP	Use for targeting
Kenya: NMSP 2019–2023				
Modelled *Pf*PR map (geostatistical modelling)	Multiple surveys and studies combined with environmental data	Second- level administrative division (sub-counties)	To show the epidemiological stratification: endemic areas (lake and coast), seasonal malaria transmission areas, malaria epidemic prone areas (western highlands of Kenya) and low risk malaria areas	Maps were used to identify epidemic and epidemic-prone areas where LLINs were to be delivered by mass distribution and routine channels at ANC; to identify zones where to implement IRS (lake endemic areas) and IPTp (lake and coastal endemic regions); and to identify zones where installing buffer stocks of case management commodities and IRS (epidemic prone areas) was appropriate
Malawi: NMSP 2017–2022				
Descriptive incidence map (cases per 1000 population)	Routine HIMS 2011–2015	Second-level administrative division level (Districts)	To show variation in incidence across districts and decline in incidence from 2011 to 2015	Maps were used to identify highly endemic districts where to implement IRS interventions
DRC: NMSP 2016-2020				
Descriptive *Pf*PR map	DHS survey 2013–2014	First-level administrative division (by the 26 new provinces created in late 2015)	To show the malaria pre-elimination, control-consolidation and control intensification areas	A maps was used to identify areas where LLINs were to be additionally distributed through schools (areas with prevalence > 30%, also defined as tropical regions) and areas where to implement IRS (in pre-elimination and urban areas in North Kivu and Kinshasa)

Participants in Malawi and DRC perceived targeting to be associated with limited resources, while in Kenya participants felt the use of a targeted approach was primarily to increase efficiency and value for money in malaria control.

In DRC, with severe resource constraints, the NMCP used what they called ‘time prioritization’, as defined by a report developed by the African Leaders Malaria Alliance (ALMA) [40]. This refers to the practice of implementing malaria control interventions in high burden regions initially, whilst searching for funding to implement the interventions in additional geographical areas. As international partners explained:

“[in] DRC: *what they have done is a time*-*bound prioritization, so they covered their 2018 and 2019 LLINs campaigns and 2020 has gaps. This it is essentially an operational programmatic prioritization of your limited resources… This is just common sense decisions a government has to make*” (Partner 2, Global).

In Malawi, although LLINs and IPTp were universally implemented, targeting was perceived, by some, as a better strategy given the resource contraints and geographical variations in risk, as explained by one official:

“*We should stop actually doing the blanket interventions because it is a waste of resources in some areas where they don’t need those interventions so we need more data that can guide us to plan for targeted interventions because in Malawi we have… yes we have malaria but… all areas are not affected equally*.” (NMCP officer 6, Malawi)

However, some other stakeholders in DRC and Malawi perceived the prioritization and targeting of high impact interventions (LLINs and IPTp) not suitable in a country where the risk of malaria is high almost everywhere. They felt that universal coverage of LLINs and IPTp was the most appropriate strategy at this stage, weakening the need for P*f*PR risk maps

“*When you are a country where everywhere is highly endemic then there is not much space anyway for prioritization*.” (Partner 2, global)

Also reflected in statements by participants from DRC and Malawi:

“*There is no prioritization of provinces [region] in relation to prevalence…not at the moment.”* (Partner 14, DRC)

“*If we had universal coverage in terms of the vector control, that would be much better so that at least we reduce the incidence, after reducing the incidence then you can now try to see where can we go….[targeting*].” (Partner 14, Malawi).

### Using maps for planning of operational interventions, commodity quantification and advocacy

Malaria maps were used for purposes beyond strategic planning and targeting. These included project monitoring or planning, supply quantification, financial justification, and budget advocacy purposes.

### Guiding and justifying commodity quantification and operational interventions

NMCPs case management division and NGOs officers described the use of risk maps to guide the quantification of commodities, such as RDTs and ACT, according to the level of burden. Indeed, population at risk, number of malaria cases and deaths and malaria prevalence maps were utilized conjointly to quantify malaria commodities in a specific area.

*“[high endemicity areas] these areas are supplied differently than the others. For example in Haut*-*Uele where the endemicity is very high with seasonal upsurges, even we talked about the epidemics, the attention is different, we bring in the inputs we repositioned elsewhere*.” (Partner officer 19, DRC).

Maps were also used to justify national decisions, such as where LLINs needed to be allocated, to the sub-regional government. Malaria control interventions, such as universal LLIN distribution and IRS, are highly visible and often local governments are interested in implementing the intervention in their area, independent of the level of malaria risk. Having a malaria risk map helped the central government to justify the geographical allocation of interventions to the sub-national level.

“*Accepting like when your program tells you really we are not giving you nets, not because we don’t like your county or your county didn’t vote for government, no. It’s because the evidence … do not have the high prevalence in malaria in your county you don’t need this. And there’re now starting to actually understand this… because I think one of the question was why are you not spraying my county, why are you not giving me nets. You are there sitting in a national meeting and hearing that nets have been distributed why not my county. So having a document or having something that you can show them and tell them it’s because of one, two, three*.” (Partner officer 8, Kenya).

### Monitoring interventions and trends over time

Maps were specifically used for annual reviews and during the mid-term and final reviews of the NMSP. Malaria risk maps of consecutive years were key to showing progress and readjusting the strategy over time, as Kenyan officials explained:

“*For us at the national level we’re using that [the map] to show progress of malaria control over time*.” (NMCP officer 1, Kenya)

“*We’re able to see the map actually shrinking or is it becoming darker but I can report that actually we’re heading [in] the right direction it’s becoming lighter and lighter. …. Yeah we’ve made a lot of gains since 2010. 2007 MIS, 2010 [MIS], 2015 [MIS] we are seeing progress.*”(NMCP officer 14, Kenya).

### Resource mobilization and advocacy

The visual nature of the maps was seen to encourage their use as powerful tools for resource mobilization and advocacy, and training purposes. Respondents in Kenya and Malawi gave examples where maps were used to encourage donors or other stakeholders to provide interventions to specific areas where there were gaps.

“*If any donor comes, wants to come with any interventions, we direct him to say okay, according to the… the distribution of the burden of malaria I think you go to this location*.” (NMCP officer 15, Malawi).

Maps were used to advocate for funds at the sub-national level, in countries with some degree of devolution, and by community-based organizations to advocate for funds or for social accountability.

“*They have used that [maps]…..to advocate for the funds from the county*.” (NMCP officer 1, Kenya)

“*In 2013 they allocated 113 million shillings for malaria [in Nairobi]. So now we were questioning what did you do with this money? Which interventions did you do?*” (Partner officer 11, Kenya).

Finally, the ease with which malaria risk could be visualized was highly appreciated and often used for training purposes to catch attention of the audience.

“*When I am training them on malaria epidemiology and decision making I project for them and give them soft copy of my presentation. In this county these are the areas that you should focus your efforts more.*” (Researcher 4, Kenya).

### Factors driving the use of risk maps by decision-makers

The second key theme explored in the analysis were the factors driving the use of risk maps for strategic planning. The decision to use malaria risk maps was motivated by their availability (or potential to be developed), the technical characteristics of the maps, and the alignment of the maps with stakeholder expectations. Technical characteristics of the maps included: the nature and quality of the data from which the maps were developed and the granularity of the data; while the alignment of the maps with stakeholder expectations included a range of related factors such as: alignment with the expected malaria epidemiological situation in the country (based on eco-climatic zone, routine data, or indications from sentinel sites); knowledge, trust and perceived ownership of the data and of the process of developing the maps.

### Timely availability of maps and data for their development

Malaria risk maps were developed using either multiple *Pf*PR data points, DHS prevalence data at one point in time, or by using routine health information system data. Modelled *Pf*PR maps were available in all three countries at the time of development of the most recent NMSP, provided by the INFORM and LINK projects. In Kenya modelled *Pf*PR maps were developed around the time of the mid-term NMSP review and were incorporated in the revised strategy 2015–2019; in DRC the maps were developed in 2014 and the NMSP 2016–2020 was developed in 2015–2016; in Malawi modelled *Pf*PR maps were developed in 2014 and the new strategy 2017–2022 in 2016–2017. Timely alignment of data and maps with malaria strategic planning cycles was a key element that increased their utilization. NMSPs are developed by NMCPs and technical partners every 5–10 years and are revised as interim strategies every couples of years. The fact that the maps were developed with recent data, at the time of the NMSP revision, facilitated their utilization.

Routine health data were available in each country and by sub-national level. These data were perceived by some stakeholders as advantageous due to their being more recent and timely compared to national survey data, which are produced every 3 to 5 years. Timeliness of the monthly routine data (the period needed to send data from health facilities to the central level) was perceived as an issue by some policy makers.

### Technical characteristics and quality of the data and maps

A key concern raised about the use of maps for decision-making involved the nature, representativeness and perceived quality of the data from which the maps were developed. Stakeholders in Malawi and DRC raised concerns about a lack of transparency and clarity on the source and breadth of data in the models used to develop the maps. This meant that stakeholders felt that they were not able to judge the quality of data from which the map was developed.

“*It is the data that was included in the model that was my biggest problem …You need to choose, which is the data that you need*…” (NMCP officer 3, Malawi)

“*I think that there are surely some biases that have entered through different studies that have been taken into account*.” (NMCP officer 1, DRC).

“*Because as long as we don’t have good data, the maps will not work. The maps will not work at all*.” (NMCP officer 3, Malawi).

Conversely, routine health data were perceived as understandable and useful in indicating the distribution of malaria burden and guiding decisions despite the acknowledgement that the data was not always of good quality. Perceptions of the quality of routine data varied across countries and stakeholders.

“*We can go with the health zone routine data… as much as we can. I think that at least the routine data allows… to have a distribution variation from one area to another and the routine data still provide satisfactory information on the fact that such area is less affected than another. Okay, we know that in terms of accuracy, it is not very reliable but in terms of distribution of burdens, it is quite satisfactory. If we have surveys with satisfactory accuracy up to the next province, we work on the improvement of the routine for the provincial deployment. This is the compromise that seems reasonable to me for a gigantic country like the DRC*.” (NMCP officer 1, DRC)

“*We still have challenges with the routine data, we have challenges with the completeness, accuracy and timeliness*.” (NMCP officer 6, Malawi)

Concerns were raised about the granularity of the data and some participants questioned how representative the maps were of populations at the sub-national level. In Kenya, modelled *Pf*PR maps for the county level were available and appreciated. Modelled *Pf*PR maps with district-level resolution were available in DRC and Malawi. However, in Malawi some stakeholders either did not know of their existence or did not feel that the data used to develop the map was accurate enough to be representative at the district level.

“*Prevalence alone I think we don’t have enough [prevalence] data to come at district levels*.” (NMCP officer 15, Malawi).

DRC officers reportedly felt that the *Pf*PR data available were not sufficient to develop a representative map and as such they decided to use the most recent DHS data (2013–2014), despite it only providing provincial-level resolution, however, they have pushed to make the 2020 DHS/MIS survey representative of the 26 provinces.

“*I’ll be more comfortable with surveys at 26 provinces, at least they give me an image close to the reality …..models ….give me things that deviate from the realities*.” (NMCP officer 1, DRC).

The fact that DHS and MIS data were representative at national or at the higher sub-national levels (e.g. region or province) but not at district-level was perceived as a limitation of the national survey data. As one respondent explained:

“*Unfortunately, MIS [Malaria Indicator Survey] only gives us by region, it’s not by district. So the smallest you can go with analysis is by region. That’s one of the challenges it will have*.”(Partner officer 14, Malawi)

NMCP officers and other stakeholders in Malawi reported using incidence data from the routine health information system because they perceived this to be the only way to develop a map at district level that they felt they could trust.

### Stakeholder ownership, involvement and alignment with stakeholders expectations

In Kenya, the NMCP and other stakeholders interviewed were proud to have shifted to a targeted approach for malaria interventions and appreciated the modelled *Pf*PR maps. Respondents felt a sense of ownership because their suggestions of what data was useful were included in the maps.

Stakeholders in all three countries highlighted the importance of the sense of ownership of the maps by NMCP and of engagement of researchers and technical advisors in supporting the NMCP to develop risk maps. Kenyan NMCP officers mentioned the long term (over 20 years) and daily collaboration with KEMRI- Wellcome Trust; how the researchers have consistently contributed to the TWGs by reviewing interventions, routine data, discussing changes in strategy and policy and generating and sharing new national and global evidence. By contrast, in the DRC and Malawi, despite the involvement of the Malaria Alert Centre (College of Medicine) in Malawi, there was a keen sense from the interviews that the NMCP did not feel sufficiently involved in research generally, and specifically in the development of the *Pf*PR maps. More importantly, the lack of involvement of the NMCP in the development of the *Pf*PR maps had negative implications for the use of the evidence based on the maps.

As one NGO official in Malawi explained:

“*Sometimes if you don’t involve the national program at the beginning …there is unwillingness to accept whatever comes out of your study*.” (Partner officer 17, Malawi).

However, in addition to trust in the data and legitimacy of the process, there were also indications that the choice of map used in the NMSP and other national documents could be influenced by whether what the maps showed was in alignment with what decision-makers expected to see based on other data sources or publications. In DRC, for example, the expectations of the NMCP, based on the routine data, aligned better with a map that was produced using DHS data than with the modelled map. A local officer commented:

“*The rendering of this model did not satisfy us, because it did not add to what we expected, and what we aimed at in terms of return routine field data… so finally we chose to make our stratification on the basis of the parasitic prevalence of EDS [DHS].*” (NMCP officer 1, DRC).

In Malawi, participants expressed a lack of confidence in the modelled *Pf*PR maps as they did not show the progress that was perceived to have been achieved by stakeholders based on a Roll Back Malaria (RBM) publication [41]. In that study, a multivariate analysis and the Lives Saved Tool (LiST) were used to hypothesize that malaria interventions from 2000 to 2010 had reduced mortality in children in the country, (mortality was assumed to be largely caused by malaria), which contradicted the modelled *Pf*PR maps, as explained by a researcher:

*“[the PfPR map] showed that there were no changes in in malaria prevalence in the country from 2000 to 2010…in contrast with the RBM impact series [which] showed that Malawi got a decrease prevalence and actually… Malawi was also awarded by the ALMA [African Leaders Malaria Alliance] with a prize.*” (Researcher 1, Malawi).

The data and the indicators utilized by the two studies were different and not comparable. However, it is logical given alternative versions of achievement that NMCP would be less accepting of the one suggesting no change in malaria prevalence after their scale-up of interventions. Furthermore, the interpretation of conflicting data could also be a challenge. Stakeholders in both countries mentioned that they preferred maps that showed what they expected to see, a decreased number of cases in Malawi and alignment with routine data in DRC.

## Discussion

In this study the use and perception of malaria risk maps were investigated in three low-medium to high-burden African countries. Previous studies have reviewed the use of cartography in malaria control planning [11, 42]; highlighted the crucial role played by additional data available and new techniques of modelling to tailor sub-national intervention plans [43, 44]; and described initiatives to support the use of modelled maps of malaria risk by malaria policy-makers [45]. This study explored both the ways in which the maps were used and the reasons why they were used by local officials and key stakeholders for strategic planning in the study settings.

This study found the use of maps to be broader than for strategic planning and targeting including for project monitoring or planning, supply quantification, financial justification, and budget advocacy. The study also found perceived needs, understanding and trust of the data source and process to construct the maps to be important drivers of the type of map chosen and their use.

### Drivers of use of malaria risk maps: trust and ownership in the “input” data

**Fig. 2:**
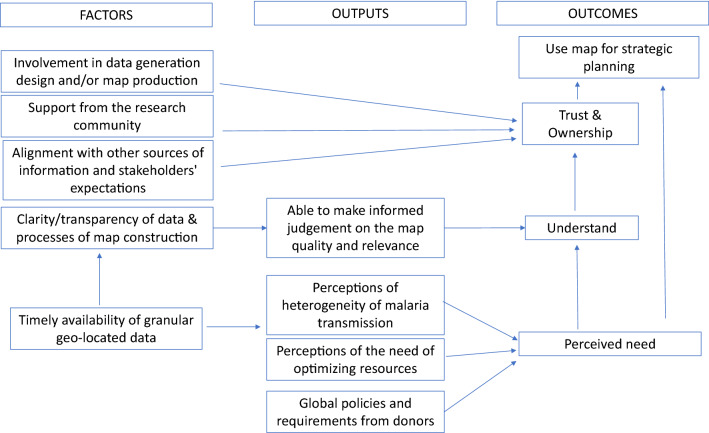
Factors influencing the type and use of malaria risk maps by malaria stakeholders

A variety of malaria risk maps were used for strategic planning and targeting to different extents across the three countries. Drivers of the use of malaria risk maps for strategic planning were: perceived need for risk maps; an understanding of what data was used to develop the map, including its limitations; and trust and ownership in the data and how it was used in the maps (Fig. 2). Perceived needs determined the preferred type of map. While the understanding of the source of data and process through which the maps were generated led to trust and ownership of the maps and ultimately to their use for strategic planning.

### Perceived needs

Perceived needs of malaria risk maps depended mainly on the perception of the level of heterogeneity of malaria in the country. For instance, malaria decision-makers interviewed in this study all recognized that Kenya had a heterogeneous distribution of the risk. Malawi and DRC had homogeneous distributions of malaria risk; however, homogeneity was perceived differently by the stakeholders according to their interests and roles. For example, in Malawi, transmission was perceived by some to differ at the sub-national level (from urban to rural and around the lake areas), whereas others described it as homogenous; in DRC the majority pointed out the high homogeneity, but others highlighted pockets of very high or very low prevalence (Kivu) or urban areas, where it would potentially be useful to develop alternative control interventions. Therefore, some stakeholders did not perceive any need for risk maps, while others expressed a need for highly granular maps defining malaria risk at the sub-district level. The potential to create maps that identify sub-national pockets of high risk of malaria is dependent upon the quality of the routine data available, prevalence data will not allow this identification. The desire for granular and timely maps along with the increasing availability and quality of malaria routine data drove some countries towards the use of routine data in the stratification of malaria, either using Test Positivity Rate or case incidence [46, 47].

The lack of perceived need for malaria risk maps by some stakeholders in countries with homogenous malaria transmission is understandable, as high impact interventions are and will be implemented universally for a significant period of time. Universal coverage among populations at greater risk is the main objective for countries such as DRC and Malawi. However, strengthening surveillance and use of data was perceived as important when allocating limited resources and to monitor progress of the control interventions..

The perception that resource allocation needed to be optimized because of limited availability, such as in DRC, or to increase the value for money of the interventions, such as in Kenya, also increased the need for targeting and developing risk maps. Finally, global policies, such as the Global Technical Strategy for malaria 2016–2030, requirements from donors, the Global Fund in particular, and the increasing support in the field for the generation and use of data (for instance through USAID funded project of Measure evaluation) would have influenced the perceived needs for production (and possibly use) malaria risk maps or stratification.

### Understanding

Understanding the meaning of malaria risk maps depended on the ability of decision-makers to make an informed judgment on the map quality and relevance. In turn, this depended on the transparency of data and how the maps were developed, and on the availability of timely and geolocated data at the desired level of granularity. The availability of timely, good quality, highly granular data would help to construct clearer maps and allow stakeholders to appreciate the quality of the map and understand what they are showing. However, high quality and timely data at high granular resolution are often not available [23] and policy makers therefore need to make decisions based on the maps using the data available or on modelled data.

Across the three countries malaria survey data and routine data were available (although to different extents and quality). Malaria surveys were generally well understood and trusted, however they were not available at the desired level of resolution and in the required timeline. Routine data were understood, although not always trusted. However, although most of the stakeholders were aware of the limitations in quality, they also considered them as the only data able “to give an idea” of the malaria risk at district level. The need to adjust for reporting rates, missing data from private sector, and the proportion of malaria that was confirmed versus suspected was recognized. But, whilst hoping that these issues would be resolved over time, policymakers were comfortable using maps based on this data because they understood the limitations and were able to make their recommendations based on this knowledge. Knowledge and understanding of the source of data and their limitations, together with knowledge and understanding of the processes of map construction were key factors in the decision-making by policy makers on what maps were appropriate to use.

### Trust and ownership

Stakeholders preferred to develop maps using data and processes that were owned, understood and trusted by the country. As noted in previous studies [22, 46, 48], the alignment with other sources of information and stakeholder expectations were considered important for stakeholders to have trust in the maps.

The NMCP in Kenya perceived the need of moving to a targeted approach to interventions, had a significant amount of data available to develop sub-national maps, understood and trusted the results of the modelled maps, and was reported by stakeholders as having allocated a budget for subsequent development of maps. Conversely, the NMCP in Malawi did not trust the results of the *Pf*PR modelling, because the modelled maps contradicted a Roll Back Malaria report, and preferred instead to use routine incidence data to develop a map at district level. The NMCP in DRC were aware of the limited number of survey points available to develop a map representative at district level and therefore they did not trust modelled maps derived from these data points. They also did not trust the quality of their routine data and chose to use the most recent DHS survey data to develop a map.

Different levels of engagement and support from the research community are likely to have influenced the development and use of a specific type of malaria risk map. The strong and long-term collaboration between the KEMRI-Wellcome Trust in Nairobi and the Kenya NMCP likely supported the perceived need to move to a targeted approach. This collaboration ensured NMCP involvement in the collection of data and definition of the type of maps to be developed, and consequently facilitated understanding of the data and the process used to develop the map. The long-term participation of the KEMRI- researchers in TWGs, and the alignment of the production of the maps with the NMSP cycle, facilitated the understanding of the process of generating modelled maps. In Malawi, the Malaria Alert Centre- College of Medicine, a nationally recognized long-term partner was involved in the development of the modelled map and profile; however, this element was not sufficient. The lack of alignment of the map with the expected results prevented the modelled map from being used.

### Policy implications

Malaria risk maps were produced and used for malaria decision-making, such as strategic planning, targeting, quantification, monitoring and advocacy. The Global Malaria Strategy 2016–2030 and the High Burden High Impact initiative highlighted the importance of increasing targeted approaches to malaria control, even in countries with a high prevalence, in order to meet the 2030 targets. However, tailored interventions may be appropriate in countries with heterogeneous risk, such as in many Sahelian countries in West Africa and the east and Horn of Africa, including Kenya. Conversely countries like Malawi and the DRC, where the risk is still very high almost everywhere, are facing multiple challenges. First, the need to ensure universal coverage must be balanced with the need to have an accurate picture of the sub-national level to identify pocket areas where a combination of additional and innovative interventions (such as vaccination, chemoprophylaxis in infant, or additional nets in school age children) may be appropriate. Second, due to limited resources there is a need to prioritize malaria control in particular areas or using specific interventions.

Modelled malaria risk maps could be one tool used to support malaria decision-making across countries. It is, therefore, important to continue supporting the development of high quality data from surveys and routine health systems that can be used to develop malaria risk maps and guide and monitor interventions. Moreover, it is key to develop maps using data and methods that policy makers understand, trust and own. The political dimension of the choice and use of data by policy makers should be recognized [49], as Newman said “*while supply of research information is important, it will only be used to inform policy if it is accessed, valued and understood by policymakers”* [50].

### Limitations

The paper analysed and compared decision-maker’s perceptions of the usefulness of malaria maps across just three countries in SSA. The three countries have different malaria epidemiological profiles (quasi homogenous in Malawi and DRC and heterogeneous in Kenya) impacting on the perceived need for P*f*PR maps to stratify malaria control interventions. Perceptions of the utility of these maps may have differed in countries with varied geographical and epidemiological contexts.

Not all of the stakeholders who were identified and contacted were available for interview. It is possible that those interviewed were more interested in malaria epidemiology and risk maps or in talking with researchers and therefore held different views to those who did not participate. All interviews were conducted by two researchers, one international and one national. It is possible that the presence of the international interviewer could have affected the way the participants replied to the questions. The interviewers were perceived as colleagues by researchers and officers of international organizations, while government officers perceived them as external figures. However, the fact that one interviewer was external to the country could have allowed the respondents to speak more freely about their perceptions.

## Conclusions

Maps were generally used to target areas of high malaria risk with specific interventions. Although national level modelled *Pf*PR maps were available in all three countries, they were not used in two of the countries. Perceived utility of malaria risks maps was associated with the epidemiological structure of the country and use was driven by perceived need, understanding (quality and relevance), ownership and trust in the data. Evidence and information to guide interventions, including malaria risk maps trusted and understood by the policy makers, are key in supporting national stakeholders to achieve their goal of effective malaria control.

## Data Availability

The datasets used and/or analysed during the current study are available from the corresponding author on reasonable request.

## Abbreviations

ALMA: African Leaders Malaria Alliance
ANC: Ante-natal care
DFID: United Kingdom Department for International Development
DHS: Demographic health survey
DRC: Democratic Republic of Congo
GF: Global Fund
IDI: In-depth interview
INFORM: Information for Malaria Project
IPTp: Intermittent preventive treatment for pregnant women
IRS: Indoor residual spraying
ITN: Insecticide-treated net
KEMRI: Kenya Medical Research Institute
LINK: LINK-Data for malaria decision-making project
LiST: Lives saved tool
LLINSs: Long lasting insecticide treated nets
LMIC: Low and middle-income countries
MiP: Malaria in pregnancy
MoH: Ministry of Health
NGO: Non-governmental organizations
NMCP: National Malaria Control Programme
NMSP: National Malaria Strategic Plan
*Pf*PR: *Plasmodium falciparum* parasite rate
PMI: President’s Malaria Initiative
RBM: Roll Back Malaria
SSA: Sub-Saharan Africa
TWGs: Technical working groups
UN: United Nation agencies
USAID: United States Agency for International Development
WHO: World Health Organization
